# Spatial Variation of the Gut Microbiota in Broiler Chickens as Affected by Dietary Available Phosphorus and Assessed by T-RFLP Analysis and 454 Pyrosequencing

**DOI:** 10.1371/journal.pone.0143442

**Published:** 2015-11-20

**Authors:** Maren Witzig, Amelia Camarinha da Silva, Rebecca Green-Engert, Katharina Hoelzle, Ellen Zeller, Jana Seifert, Ludwig E. Hoelzle, Markus Rodehutscord

**Affiliations:** Institut für Nutztierwissenschaften, Universität Hohenheim, Stuttgart, Germany; Free University of Bozen/Bolzano, ITALY

## Abstract

Molecular fingerprinting and sequencing based techniques have been widely used to characterize microbial communities. Terminal restriction fragment length polymorphism (T-RFLP) and 454-pyrosequencing were used to determine the microorganisms present in the different sections of the chicken gastrointestinal tract (GIT) (crop, jejunum, ileum and caeca). Broilers fed with diets differing in phosphorous (P) and calcium (Ca) as well as in phytase levels were used to study the microbiota of the upper and lower part of the GIT. A database with terminal restriction fragments (T-RF) of the most important organism present in the different gastrointestinal sections was constructed. The analysis revealed a distinct microbial assemblage on each section. Regardless of the diet, crop, jejunum and ileum were mainly colonized by *Lactobacillaceae*, and caeca were the most diverse site. The correlation between *Lactobacillus crispatus* and *L*. *reuteri* was positive in the crop, but negative in the jejunum. In crop samples, higher P and Ca levels led to a shift in the abundance of *L*. *reuteri* and *L*. *crispatus* to *L*. *salivarius* and *L*. *taiwanensis* whereas in the ileum supplementation of phytase favored *L*. *salivarius* and *L*. *taiwanensis* but resulted in decreased abundance of *L*. *crispatus*. Both methods were correlating significantly, being T-RFLP a reliable fingerprinting method to rapidly analyze large numbers of samples in a cost-effective and rapid manner. Results are easy to interpret with no need of deep bioinformatics knowledge and can be integrated with taxonomic information.

## Introduction

The microbiota colonizing the gastrointestinal tract (GIT) of chickens plays an important role in shaping health and productivity of the host animal [1] and is also involved in protection from pathogens, detoxification, modulation of the immune system and breakdown of feed components [2]. Species within this complex ecosystem differ in their substrate preferences and growth requirements thus are affected by the chemical composition of the digesta that is determined by the GIT section as well as by the diet composition [3]. Dietary factors such as type and content of carbohydrates and proteins, and different feed additives (e.g. probiotics, organic acids and feed enzymes) have been recognized to alter the gut microbiota in chickens [4, 5]. Moreover, variation in supplementation of minerals such as calcium (Ca) and phosphorus (P) has been shown to modulate the microbiota of rats [6] and pigs [7].

Dietary P and Ca content also affects the composition of the microbial community in the GIT of broiler chickens [8, 9]. P in plant feedstuffs is mainly present as phytate-bound P and phosphate release from phytate involves the enzyme phytase and other phosphatases. Phytases are present in dietary plant feeds [10] and to a limited extent in the mucosa of chickens [11, 12]. Phytases also can be produced by different intestinal bacteria [13]. However, some part of dietary phytate remains non-digested in the intestine of broiler chickens. Therefore, to meet the animal’s P requirements, mineral P and microbial phytases are usually added to broiler diets. These supplements affect phytate hydrolysis and P availability in the GIT [14, 15]. Amongst changes of dietary or mucosal phytase activity this may be mediated by shifts within the bacterial community composition. Studies investigating the effect of mineral P and phytase supplementation on the microbiota of chickens are rare and restricted to the ileum and/or caeca [8, 9, 16]. However, the main activity of dietary phytases can be expected in the upper GIT [17] and bacteria with a high potential to degrade phytate were found in the upper GIT [13].

Culture-independent molecular-based methods have been developed for characterization of microbial communities in different habitats. Two major approaches are community fingerprinting and sequencing-based techniques [2]. A well-established molecular fingerprinting technique of 16S rRNA gene-based analysis of microbial communities within the GIT of chickens is the terminal restriction fragment length polymorphism (T-RFLP) [18–20]. Nevertheless, a significant shift towards high-throughput next-generation sequencing methods such as 454 pyrosequencing for microbial communities characterization, is evident in the last years [2]. Despite being more affordable than ever, generating large-scale, well-replicated microbial diversity investigations with next-generation sequencing methods remains an expensive and time-consuming option [21]. Thus, several studies aimed to compare microbial community structures using T-RFLP analysis and 454 pyrosequencing. Choe and colleagues detected different bacteria in biofilms from urinary catheters depending on the use of either T-RFLP or pyrosequencing [22]. Brugger and colleagues observed a different sensitivity of both methods for discrimination of different bacterial species in the nasopharyngeal space [23]. In contrast, in studies being conducted on soil, aquifer, human feces or rumen samples both methods correlated well and gave similar results regarding the most dominant members within microbial communities [21, 24–26]. Studies in which both T-RFLP and pyrosequencing were concurrently used for characterizing the microbiota of the chicken’s GIT are not available in literature. Nordentoft and colleagues used both methods to describe the microbiota of laying hens housed in different cage systems, but the approaches were applied in a more complementary than concurrently way [27]. Thus, the aim of the present study was to investigate the sensitivity of the fingerprinting method T-RFLP against 454 pyrosequencing to compare microbial communities along the GIT of broiler chickens fed with diets differing in mineral P and Ca levels as well as in supplementation of microbial phytase.

## Material and Methods

### Ethic statement

The animal experiment was carried out in the Agricultural Experiment Station of Hohenheim University, location Lindenhöfe in Eningen (Germany), in strict accordance with the German Animal Welfare legislation. All procedures regarding animal handling and treatments were approved by the Animal Welfare Commissioner of the University.

### Birds, diets and sampling

Samples for bacterial community analysis were obtained from an animal experiment described in detail by Zeller *et al*. [15]. In brief, a 2x3 factorial arrangement of dietary treatments was used. Two corn-soybean meal-based grower diets contained P only from plant sources (BD-) or additionally supplemented P from monocalcium phosphate (MCP), (BD+). Treatments consisted of the BD- and BD+ supplemented with 0, 500 or 12,500 FTU of an *E*. *coli* phytase/kg of feed.

Broiler chicken hatchlings (strain Ross 308) were allocated to floor pens, 20 birds each. Animals were fed with a commercial starter diet until 15 days of age; this diet contained 0.90% Ca, 0.65% tP, 22.0% crude protein, 6.8% ether extract, 12.6 MJ ME/kg, and 606 FTU/kg 3-phytase (EC 3.1.3.8, 4a E1600). At day 15 of age animals were switched to experimental diets. The six dietary treatments were randomly assigned to the pens (six pens per diet) and treatment diets were fed for 10 days and were offered for *ad libitum* consumption. Samples for microbial analysis were taken on day 25 of age from four chickens per pen and six pens per diet. Birds were euthanized by carbon dioxide asphyxiation following anesthesia in a gas mixture and different segments of the digestive tract were dissected to collect luminal digesta from the crop, jejunum (second half of the intestine between gizzard and Meckel’s Diverticulum), the distal two-thirds of the ileum (from Meckel´s Diverticulum until 2 cm before the ileocaecal junction) and the two caeca. Luminal digesta samples of each segment were pooled on a pen basis and stored at -80°C.

### 454 pyrosequencing of 16S rRNA gene amplicons

Extraction of genomic DNA from luminal digesta samples was carried out using the QIAamp DNA Stool Mini Kit (Qiagen, Hilden, Germany) according to the manufacturer’s instructions. The quality and purity of DNA extracts were determined spectrophotometrically (NanoDrop UV-Vis spectrophotometer 2000c, Thermo Fisher Scientific) and checked by agarose gel electrophoresis. DNA concentrations were measured fluorometrically by using the Qubit 2.0 fluorometer and Qubit dsDNA BR Assay Kit (Life Technologies, Darmstadt, Germany).

Three of the six DNA extracts per treatment and GIT section showing the best DNA quality and quantity were used for further analysis. The microbial community composition of a pooled DNA extract of the chosen replicates per treatment and section was amplicon pyrosequenced using 27F and 1492R [28] primers and Roche GS FLX++ technology (Eurofins Scientific).

Sequenced 16S rRNA gene amplicons were processed using Mothur software [29]. Sequences were quality filtered by excluding reads that had an average quality score lower than 20, a total length of less than 400 base pairs (bp), any primer or barcode mismatch, more than 10 homopolymer stretches and an N character. Sequences were sorted regarding their barcodes and primers and barcodes were trimmed from each read. Sequences were aligned and checked for chimeras using uchime and clustered into operational taxonomic units (OTU) at ≥ 97% similarity within Mothur [29]. Low abundance OTU (< 0.0004% of total reads) were removed. A total of 969 phylotypes were taxonomically assigned using the naïve Bayesian RDP classifier [30]. Sequences were submitted to European Nucleotide Archive under the study accession number PRJEB9198. All samples comprised more than 6.825 sequence reads, where the mean number of sequences per sample was 9.400±353, totaling 225.601 usable sequences reads.

### T-RFLP profiling of 16S rRNA gene amplicons

16S rRNA gene fragments from the same pooled DNA samples as used for 454 pyrosequencing were amplified by polymerase chain reaction (PCR) using the universal bacterial primers 27F labeled at the 5′ end with 6-carboxyfluorescein (6-FAM) and 1492R [28]. PCR was performed in a total volume of 50 μl containing 10 μl of 5×PCR-Mastermix (Bio&SELL, Feucht, Germany), 0.4 μM of each primer (Biomers, Ulm, Germany), 2–15 μl of template DNA and nuclease free water (Qiagen). PCR from crop, jejunum and ileum samples were run in quadruplicates and for caecal samples in duplicates. An initial denaturation at 94°C for 4 min, followed by 30 cycles of denaturation at 94°C for 45 s, annealing at 58°C for 60 s, elongation at 72°C for 2 min and a final extension step at 72°C for 20 min was applied. Amplification products were purified using the Double Pure Kombi Kit (Bio&SELL) and fluorometrically quantified as described before.

70 ng of the purified PCR products were digested overnight using 5U of *Hae*III (New England BioLabs, Frankfurt, Germany) as according to manufacturer instructions and digests were purified using Sephadex (illustra Sephadex G-50 Fine DNA Grade, GE Healthcare, Little Chalfont, UK).

Separation of fragments was carried out by capillary electrophoresis on a 3130 Genetic Analyzer (Applied Biosystems, Darmstadt, Germany) using an internal size standard (MapMarker X-Rhodamine Labeled 50–1500 bp, BioVentures, Murfreesboro, TN, USA). Fluorescent signals of terminal restriction fragments (T-RF) were extracted in the range of 50–1000 bp using the Gene Mapper V4.1 software (Applied Biosystems) and normalized according to Abdo and colleagues [31].

In addition, each DNA extract used for that pool was processed separately as previously described and analyzed by T-RFLP as single replicate (n = 3), to give a better insight of bacterial community for each dietary treatment.

### Cloning and sequencing of 16S rRNA gene amplicons for identification of T-RFs

DNA extracts of all six treatments were pooled per GIT section (crop, ileum, caeca) and amplified as described above except that the forward primer was not fluorescently labeled. PCR products were purified with the MinElute PCR Purification Kit (Qiagen) and cloned using the TA Cloning Kit TOP10F (Life Technologies). Ninety-six clones per section were screened by amplified ribosomal DNA restriction analysis (ARDRA) using the restriction enzyme *Afl*III (BioLabs) and sequenced on an ABI 3370xl Genetic Analyzer (Applied Biosystems, Weiterstadt, Germany). Clones were taxonomically assigned using the naïve Bayesian RDP classifier [30]. Finally, 25 different clones for crop, 24 for ileum and 47 for caeca were chosen for sequencing and clones of interest were analyzed by T-RFLP as previously described. Sequences were submitted to GenBank under the accession number KP780094-KP780131.

### Statistical analysis

Two multivariate data-sets comprising the relative abundance of each T-RF and the percentage distribution of each phylotype across each sample were analyzed using PRIMER (v.6.1.6, PRIMER-E, Plymouth Marine Laboratory, UK) [32]. Sample-similarity matrices were generated using the Bray-Curtis coefficient [33] and the bacterial community structures were explored by principal coordinate analysis (PCoA) [32]. Clusters were superimposed over the PCoA plot to represent the percentage similarity within a group of samples. A similarity profile permutation test (SIMPROF) was used to seek for statistically significant clusters [34]. Statistical comparisons between different gut sections and diets were made using analysis of similarity (ANOSIM) with 999 permutations [32]. The RELATE routine in Primer 6 [32] was used to quantify the pattern match between the two resemblance matrices generated from the pooled T-RFLP and 454-pyrosequencing samples. Rho values were considered significant if less than 5% of 9999 randomly permutated were greater than the real rho value. Phylotype richness and diversity were explored using univariate measures through PRIMER [32]. Pielou’s evenness (J') is an equitability measure, which expresses how evenly the sequence reads are distributed among the OTUs and was calculated as J' = H'/H'max = H'/logS. Shannon diversity (H’) was calculated as H' = -Σi pi ln(pi), where pi is the relative abundance of the ith OTU. Pearson correlation coefficient (999 permutations) was used to measure the occurrence of a pair of species sharing the same niche in relative proportions using PRISM 6 (GraphPad Software, CA). Positive and negative correlations were considered significantly different if *p* < 0.05.

Draftsman plots charting the relative abundance of one species-of-interest against another were used to visualize negative and positive correlations between species [35].

## Results

### Microbial community structure along the GIT of broilers

The total number of OTUs and T-RFs detected in digesta samples regarding each dietary treatment and GIT section as well as Shannon diversity and Pielou’s evenness values are given in S1 Fig and S1 Table. The values showed a low diversity of the samples from jejunum and ileum, medium diversity for crop samples and a high diversity for caeca samples.

**Fig 1:**
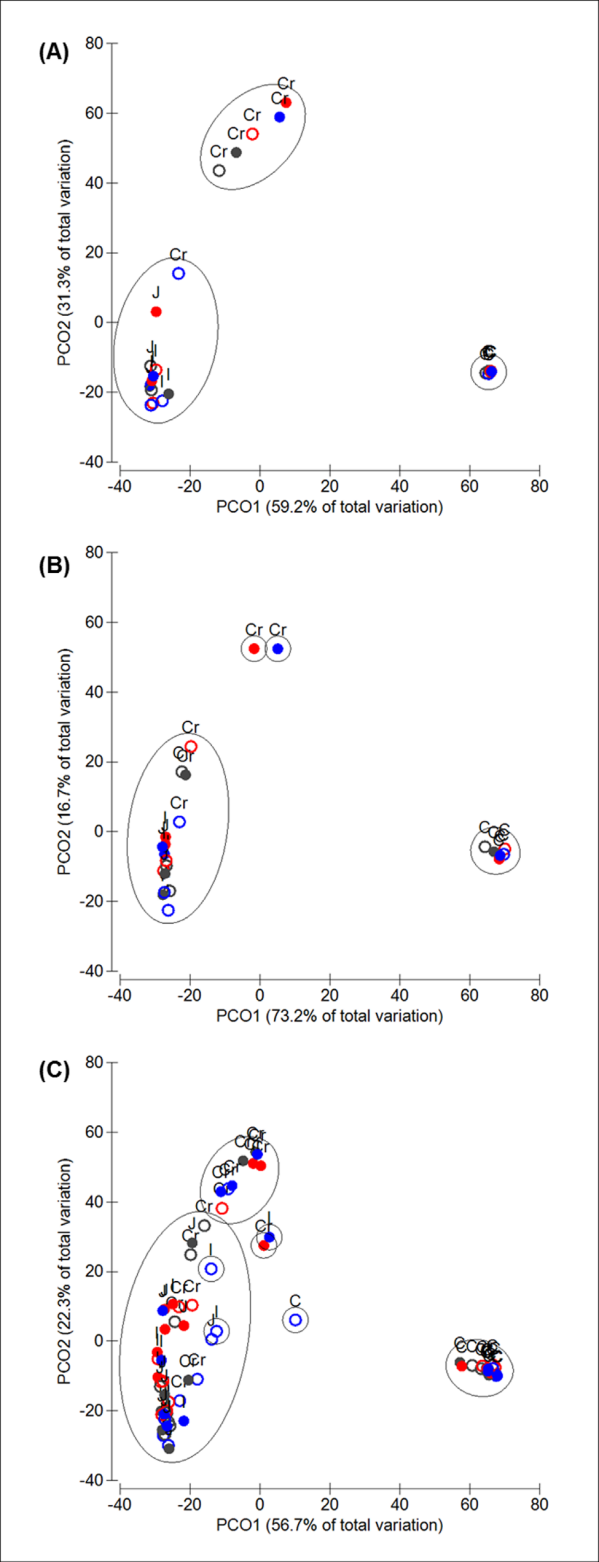
Principle coordinate analysis (PCoA) depicting the shifts in bacterial communities among the gastrointestinal tract of broilers fed with different diets varying in supplementation of monocalcium phosphate: BD- (open circles) and BD+ (close circles) and different levels of phytase: 0 (black), 500 (red), 12,500 (blue) FTU/kg feed, as detected by 454-pyrosequencing (A) and T-RFLP of pooled samples (B) and single replicates (C). Clusters represent samples sharing more than 50% similarity of their bacterial communities.

Exploring the global bacterial community structure using PCoA revealed a clear separation of bacterial communities found in caecal samples compared to those from crop, jejunum and ileum irrespective of the methodological approach (Fig 1A, 1B and 1C). Further variation of samples was associated with a shift of the bacterial community composition from crop samples to those from the small intestine. Pyrosequencing results showed a second distinct cluster with 50% similarity formed by most of the crop samples and a third one with jejunum and ileum samples (Fig 1A). T-RFLP PCoA of pooled samples showed a cluster with 50% similarity of crop samples without supplementation of MCP and all jejunum and ileum samples. Crop samples supplemented with MCP clustered together at 50% similarity (Fig 1B). Similar results were observed for T-RFLP of single replicates (Fig 1C).

ANOSIM was used to test for significant differences in the bacterial community across all the sections. A significant difference of global bacterial profiles along the different sections of broiler’s GIT was observed for all three types of analyses (454 pyrosequencing: *R* = 0.756, *p* = 0.001; T-RFLP pooled: *R* = 0.716, *p* = 0.001; T-RFLP single: *R* = 0.641, *p* = 0.001). An effect of MCP or phytase addition to the diet was not detected, in any of the cases.

**Fig 2:**
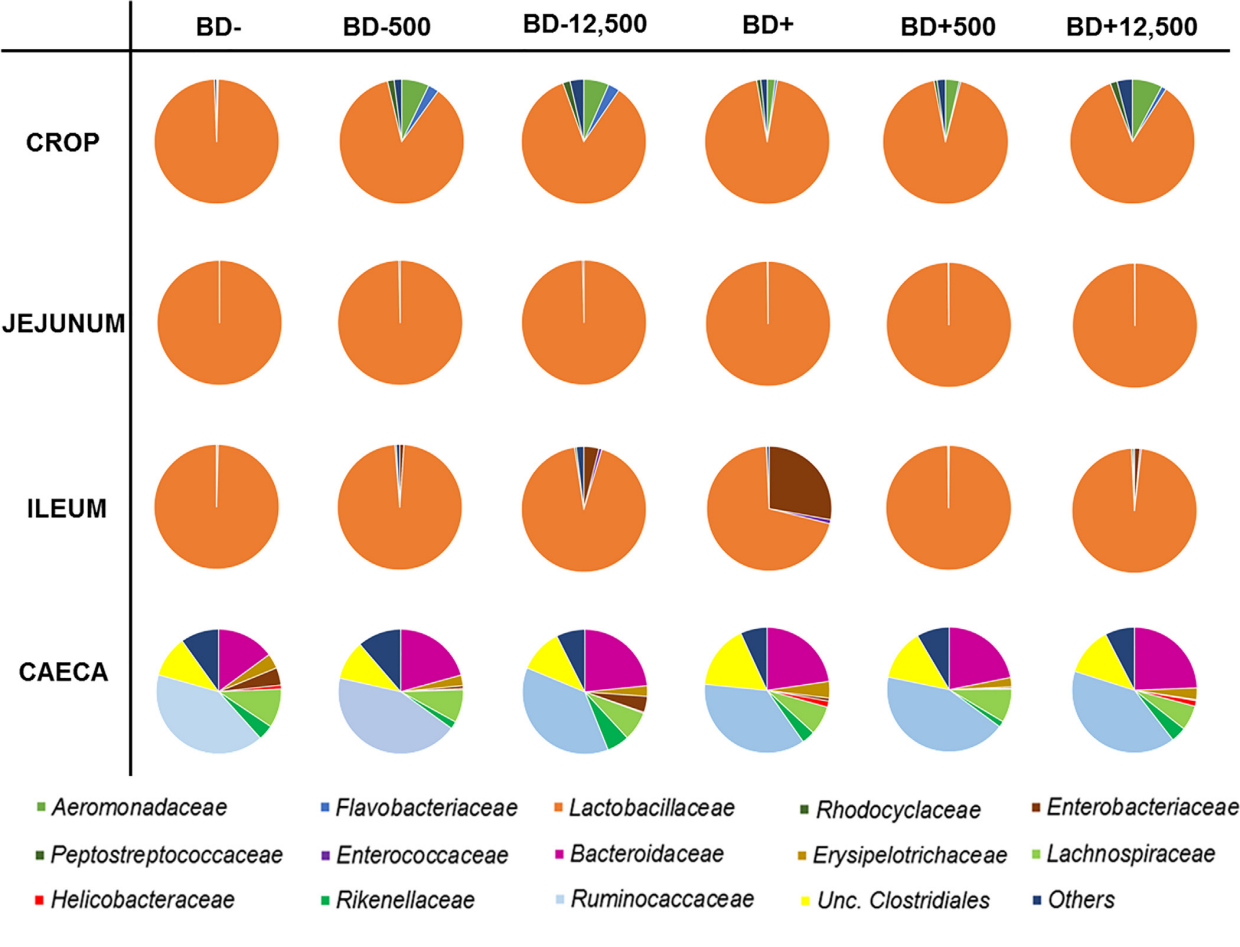
Microbiota taxonomy of GIT of chicken fed with different diets using pyrosequencing data. **Relative abundance of bacterial families within the crop, jejunum, ileum and caeca.** Diets differed in supplementation of monocalcium phosphate (BD-/BD+) and phytase (0, 500, 12,500 FTU/kg feed).

Throughout the four GIT sections investigated, bacteria from eight different phyla were identified by 454 pyrosequencing with *Firmicutes*, *Proteobacteria* and *Bacteroidetes* being the most abundant ones and represented by 867, 34 and 26 out of 969 OTUs, respectively. *Firmicutes* dominated in all four GIT sections. In jejunum samples more than 99.8% of the sequences found were affiliated to this phylum. *Proteobacteria* were observed in the crop and in the ileum in relative abundances lower than 27% and together with *Firmicutes* more than 97% of total bacterial community was covered. In caecal samples a considerable contribution of *Bacteroidetes* (>18%), *Tenericutes* and *Proteobacteria* (1–5%) to the bacterial community composition was observed. At family level digesta samples from crop, jejunum and ileum were mainly colonized by species belonging to *Lactobacillaceae* family while phylotypes belonging to *Ruminococcaceae*, *Bacteroidaceae*, uncultured *Clostridiales*, and *Streptococcaceae* (circa 80% of total community; Fig 2) were detected in caeca.

The most abundant OTUs, with their relative abundance on each section per diet are listed in Table 1 and S2 Table. The phylotypes contributing significantly to the observed differences between GIT sections were closely related to *Lactobacillus crispatus*, *L*. *salivarius*, *L*. *taiwanensis*, *Bacteroides fragilis*, *L*. *aviarius*, *Shigella flexneri*, *Aeromonas sharmana* and *L*. *vaginalis*. Sequencing of clone libraries showed a similar phylogenetic picture (Table 2). While *L*. *salivarius*-type phylotypes were the most abundant species found in the crop (>46%), sequences obtained from the small intestine mainly represented *L*. *crispatus-*type species (>81% for jejunum, >77% for ileum). In the crop this phylotype was detected on a relative average abundance of 19% and was less abundant in caeca (<0.4%). A similar trend for both lactobacilli were found by T-RFLP, where T-RF designated to *L*. *salivarius* decreased from crop to caeca and T-RF designated to *L*. *crispatus* made up 36% in the crop, 78% in the small intestine and only 2% in the caeca. The second important phylotype occurring in the small intestine, *L*. *aviarius* (>7% of all OTU), was also of minor importance in crop and caeca (<0.2% of all OTU). Differences between both sections of the small intestine were mainly explained by the decreased abundance of *L*. *crispatus-* and the increased abundance of *Shigella flexneri-*type species in the ileum when compared to the jejunum. Moreover, a continuous drop of the abundance of *L*. *taiwanensis-* and *L*. *vaginalis-*type phylotypes could be detected from crop to caeca significantly contributing to observed differences between sections. This was confirmed by T-RFLP analysis showing a decrease of T-RF designated to *L*. *taiwanensis* and *L*. *vaginalis/L*. *reuteri* from the crop to the ileum. Sequences being most abundant in caecal samples (>2.5%) showed a very low abundance in the upper GIT (<0.07%) and were classified as *B*. *fragilis*-, *Alistipes finegoldii*-, *Pseudoflavonifractor capillosus*- and *B*. *ovatus*-type species. The first three mentioned phylotypes could be identified by T-RFLP (Table 2) and three further T-RFs 195, 308, 315 with more than 2.5% abundance contributed to significant differences between caecal samples and those of the upper GIT but could not be identified by cloning. More clones were obtained from the caeca samples, however they showed a low identity with sequences available on the database and were not taken into consideration in this work. Mantel test showed a significant correlation between the results obtained with 454 pyrosequencing and T-RFLP of pooled samples (*R* = 0.92, *p* = 0.001). Moreover, SIMPER analysis for T-RFLP of the three single replicates per treatment revealed the same species contributing to differences between crop, jejunum, ileum and caeca as for T-RFLP of pooled samples.

**Table 1:** List of the 30 most abundant OTUs found among the GIT of broilers detected by 454-pyrosequencing, their average abundance in different sections and sequence similarity to closest reference strains in GenBank.

OTU	Average abundance (%)				Closest reference strain (Genbank accession no.)	RDP^a^score
	Crop	Jejunum	Ileum	Caeca		
1	19.29	81.93	77.67	0.34	*Lactobacillus crispatus* (AF257097)	0.92
2	46.47	3.42	4.94	0.04	*Lactobacillus salivarius* (AF089108)	0.92
3	18.21	6.13	1.04	0.02	*Lactobacillus taiwanensis* (EU487512)	0.91
4	0.02	<0.01	0.07	16.86	*Bacteroides fragilis* (CR626927)	0.96
5	0.11	7.29	7.95	0.12	*Lactobacillus aviarius* (M58808)	0.87
6	0.17	0.03	5.60	1.80	*Escherichia/Shigella flexneri* (X96963)	0.89
7	0.01	0	0.03	4.15	*Paenibacillus amylolyticus* (D85396)	0.42
8	0.01	<0.01	<0.01	3.43	*Alistipes finegoldii* (AY643083)	0.90
9	<0.01	0	<0.01	2.92	*Pseudoflavonifractor capillosus* (AY136666)	0.76
10	3.75	0.13	0.09	0	*Lactobacillus vaginalis* (AF243177)	0.90
11	<0.01	0	0.04	2.77	*Bacteroides ovatus* (AB050108)	0.80
12	3.77	0.01	0.03	0	*Aeromonas sharmana* (DQ013306)	0.82
13	<0.01	0	<0.01	2.59	*Pseudoflavonifractor capillosus* (AY136666)	0.67
14	<0.01	0	<0.01	2.11	*Clostridium orbiscindens* (Y18187)	0.82
15	0	0	<0.01	1.82	*Bacillus thermocloacae* (Z26939)	0.43
16	<0.01	0	<0.01	1.63	*Bacteroides thetaiotaomicron* (AE015928)	0.91
17	<0.01	0	<0.01	1.57	*Oscillibacter valericigenes* (AB238598)	0.60
18	0.01	0	0.02	1.40	*Eubacterium desmolans* (L34618)	0.78
19	<0.01	0	<0.01	1.17	*Clostridium termitidis* (FR733680)	0.46
20	<0.01	0	0.01	1.13	*Clostridium bolteae* (AJ508452)	0.71
21	<0.01	0	<0.01	1.11	*Clostridium spiroforme* (X75908)	0.86
22	0.01	0	<0.01	1.14	*Clostridium leptum* (AJ305238)	0.39
23	<0.01	0	0	1.03	*Phascolarctobacterium faecium* (X72865)	0.69
24	1.13	0.08	0.06	<0.01	*Lactobacillus reuteri* (L23507)	0.73
25	0	0	0	0.99	*Spiroplasma chinense* (AY189126)	0.39
26	0	0	0	0.89	*Anaerotruncus colihominis* (AJ315980)	0.60
27	0.01	0	<0.01	0.86	*Oscillibacter valericigenes* (AB238598)	0.60
28	<0.01	0	<0.01	0.82	*Butyricicoccus pullicaecorum* (EU410376)	0.44
29	<0.01	0	<0.01	0.87	*Eubacterium tortuosum* (L34683)	0.55
30	<0.01	0	<0.01	0.89	*Clostridium methylpentosum* (Y18181)	0.56
**Total**	**93**	**99**	**98**	**54**		

^a^Ribosomal database project

**Table 2:** Predicted and observed terminal restriction fragments (T-RFs) in base pairs (bp) of clones of interest detected in clone libraries and associated T-RFs obtained from T-RFLP of pooled samples after digestion with *Hae*III.

Clone	Genbank accecssion no.	Closest reference strain (Genbank acc. no.)	RDP score	Predicted T-RF^a^	Observed T-RF	Pseudo-T-RF I^b^	Pseudo-T-RF II^b^	Observed T-RF pooled samples
K11	KP780124	*Lactobacillus reuteri (L23507)*	0.83	67	62	336		62
K1	KP780095	*Lactobacillus vaginalis (AF243177)*	0.95	66	62			62
C14	KP780096	*Faecalibacterium prausnitzii (AJ413954)*	0.80	185	183	317	332	183
C26	KP780098	*Clostridium amygdalinum (AY353957)*	0.80	237	234			235*
C37	KP780099	*Pseudoflavonifractor capillosus (AY136666)*	0.81	239	236			236
C6	KP780102	*Clostridium lactatifermentans (AY033434)*	0.85	242	241			241
K5	KP780121	*Lactobacillus crispatus (AF257097)*	0.98	243	243			n.o.
I3	KP780103	*Lactobacillus crispatus (AF257097)*	1	246	244			244
C44	KP780104	*Bacteroides ovatus (AB050108)*	0.87	261	260	267		260
C74	KP780105	*Bacteroides fragilis (CR626927)*	0.96	263	262	851		261*
C110	KP780131	*Helicobacter pullorum (AY631956)*	0.95	270	267			266*
C64	KP780109	*Clostridium saccharolyticum (Y18185)*	0.80	273	271			271
C75	KP780128	*Eubacterium desmolans (L34618)*	0.82	275	275			n.o.
K3	KP780111	*Lactobacillus salivarius (AF089108)*	0.96	279	277	321		276*
I46	KP780125	*Lactobacillus aviarius (M58808)*	0.90	280	277			276*
K17	KP780120	*Lactobacillus salivarius (AF089108)*	0.96	278	278	323		278
K44	KP780113	*Hordeum vulgare subsp*. *vulgare (EF115541)*	0.99	295	293			293
K62	KP780114	*Lactobacillus salivarius (AF089108)*	0.94	323	321			321
K9	KP780115	*Lactobacillus salivarius (AF089108)*	0.96	323	323			322*
K12	KP780116	*Lactobacillus taiwanensis (EU487512)*	0.97	332	330			331*
K13b	KP780119	*Lactobacillus reuteri (L23507)*	0.85	336	333			333
C11	KP780117	*Bacteroides thetaiotaomicron (AE015928)*	0.98	383	381	338	339	381
C7	KP780118	*Alistipes finegoldii (AY643083)*	0.98	913	919	333		919

^a^Predicted from *in silico* digest with *Hae*III by using REPK (v.1.3) [59]

^b^Secondary peaks produced by some clones in addition to the main peak.

n.o.—not observed

^*^Observed T-RF in T-RFLP of pooled samples was assumed to represent the respective clone as run-to-run variability of fragment size is expected to be ±1bp [60].

**Fig 3:**
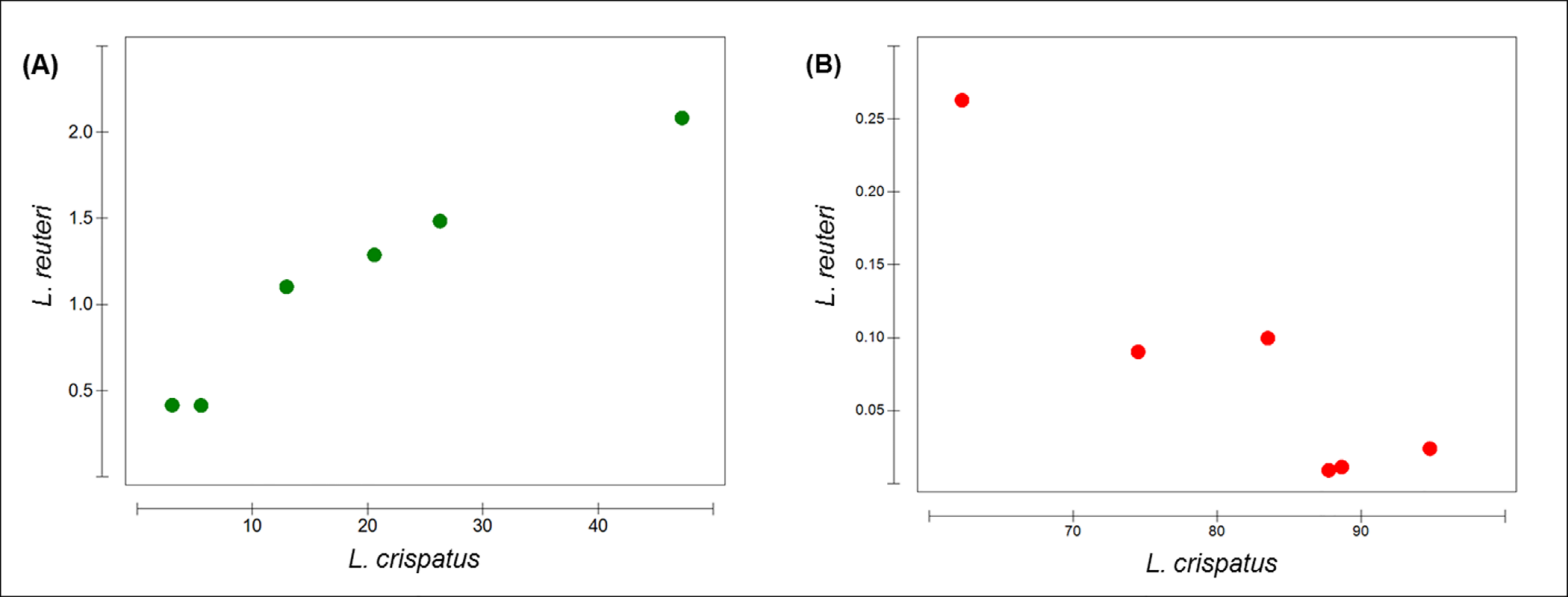
Draftsman plot showing the relative abundance (%) of one species-of-interest against another. (A) Positive correlation in crop and (B) negative correlation in jejunum between *L*. *crispatus* and *L*. *reuteri* in all six dietary treatments.

For detection of pairs of species sharing the same niche in relative proportions Person correlations were calculated (S3 Table). In crop samples *L*. *salivarius* was negatively correlated with *L*. *crispatus*, *L*. *vaginalis* and *L*. *reuteri*, however the last 3 species were positively correlated with each other (Fig 3, S3 Table). In the jejunum *L*. *crispatus* and *L*. *reuteri* were negatively correlated (Fig 3). Moreover, a higher abundance of *L*. *crispatus* was accompanied by a lower abundance of *L*. *taiwanensis* in this section (S3 Table). The resulting positive correlation between *L*. *taiwanensis* and *L*. *reuteri* observed in the jejunum was also found in the ileum. The negative correlation of *L*. *salivarius* and *L*. *vaginalis* detected in the crop was also observed in the ileum (S3 Table).

### Effect of diet on the microbial community structure within single sections of the GIT in broilers

The bacterial community structure within single sections of the GIT did not show significant effects of MCP or phytase supplementation in case of analyzing pooled samples with 454 pyrosequencing or T-RFLP (*p*>0.05) (S2 Fig). However, at family level results of 454 pyrosequencing indicated a higher abundance of *Flavobacteriaceae* in the crop digesta when no MCP was added to the diet (Fig 2, S4 Table). Moreover, supplementation of phytase led to an increasing abundance of *Aeromonadaceae* and *Flavobacteriaceae* and a decreasing abundance of *Lactobacillaceae* in the crop. No effect of MCP or phytase was detected in the jejunum. However, in the ileum, supplementation of phytase seemed to favour the abundance of *Enterobacteriaceae* while decreasing *Lactobacillaceae* in diets without MCP. The opposite was found in diets with MCP. In caecal samples *Enterobacteriaceae* were less prevalent and unclassified *Clostridiales* and *Bacteroidaceae* were more abundant when MCP was supplemented. In the absence of MCP, the addition of phytase led to a lower abundance of *Erysipelotrichaceae* and *Lactobacillaceae* and to a higher abundance of *Bacteroidaceae*.

**Fig 4:**
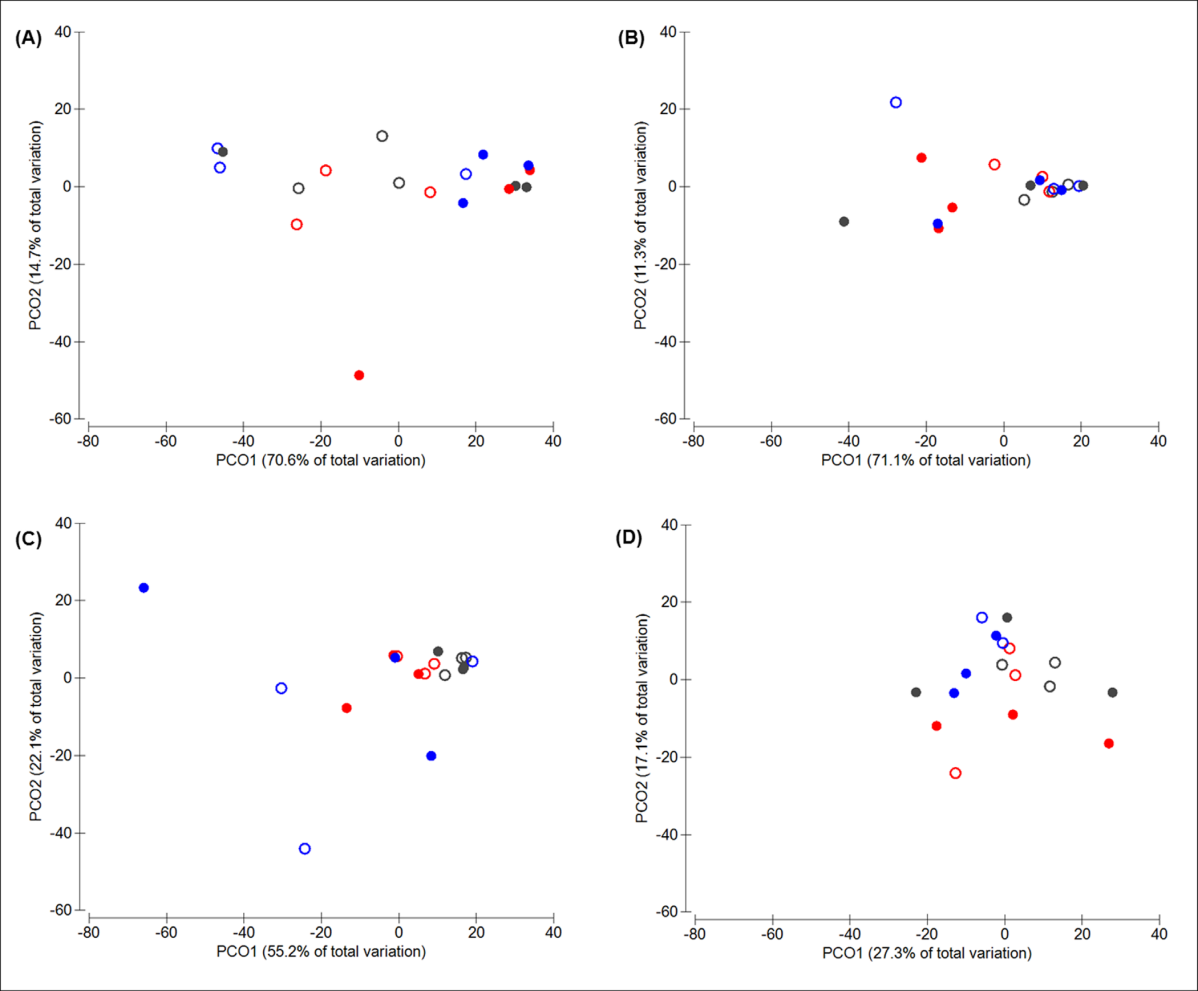
PCoA ordination of the bacterial assembly of single replicates within each section of GIT. Broilers were fed with different diets varying in supplementation of monocalcium phosphate: BD- (open circles) and BD+ (close circles) and different levels of phytase: 0 (black), 500 (red), 12,500 (blue) FTU/kg feed, and samples analyzed by T-RFLP: Crop (A), Jejunum (B), Ileum (C) and Caeca (D).

PCoA for T-RFLP of single replicates revealed that more than 70% of total variation between bacterial community structures in crop samples are explained by PCo1 indicating a strong effect of MCP (Fig 4). This was also confirmed by ANOSIM (*R* = 0.291, *p* = 0.009). However, no effect of phytase was detected in crop samples. In the digesta of the jejunum no effects of MCP or phytase on bacterial community structures was found. In the case of the ileum a significant shift of the bacterial community composition with addition of phytase was observed as more than 55% of total variation was explained by PCo1 (Fig 4). This was also confirmed by ANOSIM (*R* = 0.229, *p* = 0.001). For caecal samples no significant effect of both dietary factors were observed but a possible trend for a change in the bacterial community structure with addition of phytase became evident by PCoA (Fig 4) as well as by ANOSIM (*R* = 0.136, *p* = 0.06).

SIMPER analysis for T-RFLP of single replicates indicated a higher abundance of *L*. *reuteri*/*L*. *vaginalis-* (T-RF 62) and *L*. *crispatus*-type species (T-RF 244) in crop samples in the absence of MCP whereas for diets with addition of MCP *L*. *salivarius*- (T-RF 278 and 321) and *L*. *taiwanensis*-type phylotypes (T-RF 331) seemed to be more abundant. For the ileum increased abundances of species affiliated to *L*. *salivarius* (T-RF 278, 321 and 322) and *L*. *taiwanensis* (T-RF 331) with supplementation of phytase was shown whereas for diets without phytase *L*. *crispatus*-type species (T-RF 243 and 244) were favored.

## Discussion

In the last decade 454-pyrosequencing became one of the most used method to depict microbial communities from a vast set of habitats [21, 36, 37]. However, it is still a costly technique if a large amount of samples needs to be analyzed. T-RFLP is an established fingerprinting technique that can characterize microbial communities rapidly and cost-efficiently [21]. To identify phylotypes by T-RFLP, clones with known sequences were used for an *in silico* and experimental restriction digestion. This enabled the assignment of different species and revealed the well-known problem of pseudo-T-RFs, which were shown for 12 of the clones here. These pseudo-T-RFs were probably caused by a partial amplicon digestion [38, 39]. Further work should be done in order to identify three unknown T-RFs detected in the caeca samples in abundances higher than 2.5%, which were not covered by existing clone libraries. Several studies with different sample sources have shown that T-RFLP is a suitable method that coupled with sequencing information gives a reliable insight into community profile [40–42]. Results from the present study confirmed that 454 pyrosequencing and T-RFLP have a strong correlation and revealed similar global results analyzing the 16S rRNA gene. As previously demonstrated with soil [21], nasopharyngeal [23] and rumen [24] samples, pyrosequencing had a better insight into the bacterial community. Nevertheless, T-RFLP was also able to distinguish the different sections of the GIT in broiler chickens and the same dominant species per sample were detected as those detected by pyrosequencing.

Independently of the diet, lactobacilli were the dominant colonizers of the crop, where a negative correlation between *L*. *salivarius* and *L*. *crispatus* was observed. In presence of in-feed antimicrobials *L*. *crispatus* increased its prevalence [43]. *L*. *salivarius* was highly abundant in the crop samples, its abundance decreased in the jejunum and ileum while *L*. *crispatus* became the most abundant microorganism. Previous studies have shown that *L*. *crispatus* inhibits the adhesion of avian pathogenic *Escherichia coli* [44]. Also, it has high amylase activity, which is a favorable effect on animal performance and feed conversion ratio [45]. Abbas Hilmi and colleagues have shown that *L*. *reuteri* was the most abundant lactobacillus species in the crop of 1-week old chicken decreasing its abundance in 5-week old chicken depending on the diet [46]. In the current study this microorganism was not the most abundant in the crop but its abundance decreased in diets supplemented with MCP. In addition, it showed a positive correlation with *L*. *crispatus* in the crop, but the opposite was found in the jejunum where a negative correlation was observed. In the present study, lactobacilli were detected in abundances lower than 1% in the caeca, which is in accordance with some published studies [43, 47, 48]. The caeca is the highest diverse section of the chicken GIT (9–12 fold more) and is mainly colonized by unknown or uncultured microorganisms. A great number of OTUs could not be assigned at the genus level. This might be related to the fact that most of our knowledge regarding intestinal samples came from human studies leaving other hosts not so well characterized and with absence of important information [49, 50].

Results from *in vitro* studies with ruminal bacteria have shown that microbes require a minimum of P for optimal fermentation [51]. Moreover, it was demonstrated that bacterial P and Ca assimilation and metabolic activity depend on P and Ca availability in the large intestine of pigs [52]. In the present study, a significant effect of dietary MCP or phytase was obtained by T-RFLP analysis of single replicates. In crop samples, a shift within *Lactobacillales* from *L*. *salivarius*- and *L*. *taiwanensis*- to *L*. *reuteri/L*. *vaginalis*- and *L*. *crispatus*-type phylotypes, when no MCP was added, was detected. Palacios and colleagues isolated phytase-producing lactobacilli from different sections of the GIT of broiler chickens [13]. *L*. *reuteri*, isolated from the gizzard, showed one of the highest phytate-degrading activity (600–1700 U/g protein at pH 5.0 and 7.2) [13]. Several *L*. *crispatus* strains isolated from crop and caeca of broiler chickens have also been shown to possess phytase activity [45]. The ability of utilizing phytate-bound P may explain the higher abundance of both species when animals were fed with phytate-rich diets without MCP. However, *L*. *salivarius*-type species isolated from the small intestine [13] and feces [53] of broiler chickens also had high phytate-degrading activities. Either these strains differ from those strains in the crop in their ability to degrade phytate or other factors such as changes in physicochemical properties of the digesta, play a role for the occurrence of *L*. *salivarius*-type species. Differences in pH and production of short chain fatty acids were observed in the crop of 42-day-old broiler chickens fed with low compared to adequate contents of P and Ca in diets [9]. Moreover, free Ca^++^ ions have been shown to considerably improve the attachment of *L*. *salivarius* isolated from the chicken GIT to immobilized chicken mucus *in vitro* [54] whereas the positive effect of Ca^++^ ions on *in vitro* adhesion of a *L*. *reuteri* isolate from pig feces to piglet jejunal epithelial cells was much lower than for other *Lactobacillus* sp. [55].

An effect of MCP on the bacterial community composition in the ileum or caeca of broilers was not detected by T-RFLP analysis in the present study whereas results on bacterial counts and fermentation products reported previously indicated shifts within the microbial community in these sections when feeding diets low or adequate in P and Ca [8, 9]. These different findings may result from differences in diet formulations, methodical approaches used for microbial community analysis or animal’s age.

A significant effect of phytase on the bacterial community composition was only observed in the ileum being restricted to *L*. *salivarius-*, *L*. *taiwanensis-* and *L*. *crispatus-*type phylotypes. Changes in concentrations of different inositol phosphate isomers resulting from effects of phytase on phytate degradation were reported in a companion study [15]. Phytate can also exert anti-nutritional effects through formation of complexes with proteins and or minerals. Hence, supplementation of phytase is associated with positive effects on the availability of different nutrients in digesta through increased phytate breakdown [5]. However, the enzyme mechanism that caused an increase in the abundance of the *L*. *salivarius*- and *L*. *taiwanensis*-type species and the decrease of *L*. *crispatus* is unknown. An effect of phytase on the abundance of *L*. *reuteri* was not found in the ileum. This is in accordance with previous findings in pig ileum samples [56]. However, total counts of lactobacilli in the ileum of broilers were increased upon supplementation of 500 or 5000 U [8, 9].

The possible trend for a change in the bacterial community structure with addition of phytase in caeca detected in the present study is in accordance with results of previous studies that showed changes in lactobacilli and *Escherichia coli* counts (8) or production of short chain fatty acids [9] in caeca of broilers when adding phytase to diet.

Studies on the bacterial community composition in the jejunum of broiler chickens as affected by diet are rare. Geier and colleagues demonstrated an effect of indigestible carbohydrates on the composition of the *Lactobacillus* community in the jejunum of 25-day-old broiler chickens [57]. However, Torok *et al*. detected no effect of a non-starch polysaccharide-degrading enzyme on the jejunal bacterial community composition in 22-day-old broiler chickens [58]. Likewise, no effect of the MCP or phytase on the bacterial community composition in the jejunum could be observed in the present study.

## Conclusions

This is the first study showing that 454 pyrosequencing and T-RFLP can be used for analysis of the microbiota in broiler GIT. Results of both methods were highly correlated and revealed similar global results on bacterial communities found in crop, jejunum, ileum and caeca. Thus, we conclude that T-RFLP can be used as a reliable fingerprinting method to rapidly analyze large numbers of samples. Moreover, our results provide comprehensive taxonomic information on the bacterial community that is present in different sections of chicken’s GIT. To the best of our knowledge, we are the first having used such a high number of animals (72) to analyze the chicken’s microbiota considering the upper as well as the lower part of the GIT. Furthermore, this is the first report on effects of MCP and phytase on the microbiota in different sections of chicken’s GIT. Our data revealed a strong dietary effect on lactobacilli in crop and ileum samples. Shifts on different bacterial groups within the microbiota were also observed at family level. Hence, it can be concluded that MCP as well as phytase are modulators of the gut microbiota in chickens.

## Supporting Information

S1 Fig Number of OTUs (light blue) and TRFs of pooled samples (yellow) and TRFs of single replicates (Mean, SD; n = 3) (dark blue) and their respective values for Shannon diversity (H’) (circles) and Pielou’s evenness (J’) (triangles) detected for different gut sections from broiler chickens fed diets varying in monocalcium phosphate (BD-/BD+) and phytase (0, 500, 12,500 FTU/kg feed) supplementation. (TIFF)

S2 Fig Shifts in bacterial community structure within different sections of the gastrointestinal tract of broilers fed different diets as revealed by pooled samples. Comparison of PCoA plots for T-RFLP of pooled and 454-pyrosequencing results and samples per dietary treatment. Diets varied in supplementation of monocalcium phosphate: BD- (open circles) and BD+ (close circles) and different levels of phytase: 0 (black), 500 (red), 12,500 (blue) FTU/kg feed.
(TIFF)

S1 Table Number of operational taxonomic units (OTUs) and terminal restriction fragments (T-RFs) as well as Shannon diversity (H’) and Pielou’s evenness (J’) for pooled digesta samples and single replicates (Mean, *SD*; n = 3) detected for different gut sections from broiler chickens fed diets varying in monocalcium phosphate (BD-/BD+) and phytase (0, 500, 12,500 FTU/kg feed) supplementation. (DOCX)

S2 Table List of OTUs contributing for more than 92% of total bacteria present in the crop, jejunum and ileum and 50% in the caeca as detected by 454-pyrosequencing. (DOCX)

S3 Table Positive and negative correlations within crop, jejunum, ileum and caeca. (DOCX)

S4 Table Relative abundance (%) of bacterial families within the crop, jejunum, ileum and caeca. Diets differed in supplementation of monocalcium phosphate (BD-/BD+) and phytase (0, 500, 12,500 FTU/kg feed).
(DOCX)

## References

[1] Wei S, Morrison M, Yu Z. Bacterial census of poultry intestinal microbiome. Poultry Sci. 2013;92(3):671–83. 23436518 10.3382/ps.2012-02822

[2] Stanley D, Hughes RJ, Moore RJ. Microbiota of the chicken gastrointestinal tract: influence on health, productivity and disease. Appl Microbiol Biotechnol. 2014;98(10):4301–10. 10.1007/s00253-014-5646-2 24643736

[3] Apajalahti J, Kettunen A, Graham H. Characteristics of the gastrointestinal microbial communities, with special reference to the chicken. Worlds Poult Sci J. 2004;60(02):223–32.

[4] Chambers JR, Gong J. The intestinal microbiota and its modulation for Salmonella control in chickens. Food Res Int. 2011;44(10):3149–59.

[5] Kiarie E, Romero LF, Nyachoti CM. The role of added feed enzymes in promoting gut health in swine and poultry. Nutr Res Rev. 2013;26(1):71–88. 10.1017/S0954422413000048 23639548

[6] Bovee-Oudenhoven IM, Wissink ML, Wouters JT, Van der Meer R. Dietary calcium phosphate stimulates intestinal lactobacilli and decreases the severity of a salmonella infection in rats. J Nutr. 1999;129(3):607–12. 10082763 10.1093/jn/129.3.607

[7] Mann E, Schmitz-Esser S, Zebeli Q, Wagner M, Ritzmann M, Metzler-Zebeli BU. Mucosa-associated bacterial microbiome of the gastrointestinal tract of weaned pigs and dynamics linked to dietary calcium-phosphorus. PLoS One. 2014;9(1):e86950 10.1371/journal.pone.0086950 24466298 PMC3900689

[8] Akyurek H, Ozduven ML, Okur AA, Koc F, Samli HE. The effects of supplementing an organic acid blend and/or microbial phytase to a corn-soybean based diet fed to broiler chickens. Afr J Agr Res. 2011;6(3):642–9.

[9] Ptak A, Bedford MR, Swiatkiewicz S, Zyla K, Jozefiak D. Phytase modulates ileal microbiota and enhances growth performance of the broiler chickens. PLoS One. 2015;10(3):e0119770 10.1371/journal.pone.0119770 25781608 PMC4363628

[10] Weremko D, Fandrejewski H, Zebrowska T, Han IK, Kim JH, Cho WT. Bioavailability of phosphorus in feeds of plant origin for pigs—Review. Asian Australas J Anim Sci. 1997;10(6):551–66.

[11] Maenz DD, Classen HL. Phytase activity in the small intestinal brush border membrane of the chicken. Poult Sci. 1998;77(4):557–63. 9565239 10.1093/ps/77.4.557

[12] Huber K, Zeller E, Rodehutscord M. Modulation of small intestinal phosphate transporter by dietary supplements of mineral phosphorus and phytase in broilers. Poult Sci. 2015;94(5):1009–17. 10.3382/ps/pev065 25834252

[13] Palacios MC, Haros M, Sanz Y, Rosell CM. Selection of lactic acid bacteria with high phytate degrading activity for application in whole wheat breadmaking. LWT—Food Science and Technology. 2008;41(1):82–92.

[14] Shastak Y, Zeller E, Witzig M, Schollenberger M, Rodehutscord M. Effects of the composition of the basal diet on the evaluation of mineral phosphorus sources and interactions with phytate hydrolysis in broilers. Poult Sci. 2014;93(10):2548–59. 10.3382/ps.2014-03961 25085939

[15] Zeller E, Schollenberger M, Witzig M, Shastak Y, Kühn I, Hoelzle LE, et al Interactions between supplemented mineral phosphorus and phytase on phytate hydrolysis and inositol phosphates in the small intestine of broilers. Poult Sci. 2015;94(5):1018–29. 10.3382/ps/pev087 25810408

[16] Aydin A, Pekel AY, Issa G, Demirel G, Patterson PH. Effects of dietary copper, citric acid, and microbial phytase on digesta pH and ileal and carcass microbiota of broiler chickens fed a low available phosphorus diet. J Appl Poultry Res. 2010;19(4):422–31.

[17] Dersjant-Li Y, Awati A, Schulze H, Partridge G. Phytase in non-ruminant animal nutrition: a critical review on phytase activities in the gastrointestinal tract and influencing factors. J Sci Food Agric. 2015;95(5):878–96. 10.1002/jsfa.6998 25382707 PMC4368368

[18] Chee SH, Iji PA, Choct M, Mikkelsen LL, Kocher A. Characterisation and response of intestinal microflora and mucins to manno-oligosaccharide and antibiotic supplementation in broiler chickens. Br Poult Sci. 2010;51(3):368–80. 10.1080/00071668.2010.503477 20680872

[19] Geier MS, Torok VA, Guo P, Allison GE, Boulianne M, Janardhana V, et al The effects of lactoferrin on the intestinal environment of broiler chickens. Br Poult Sci. 2011;52(5):564–72. 10.1080/00071668.2011.607429 22029783

[20] Torok VA, Hughes RJ, Mikkelsen LL, Perez-Maldonado R, Balding K, MacAlpine R, et al Identification and characterization of potential performance-related gut microbiotas in broiler chickens across various feeding trials. Appl Environ Microbiol. 2011;77(17):5868–78. 10.1128/AEM.00165-11 21742925 PMC3165380

[21] van Dorst J, Bissett A, Palmer AS, Brown M, Snape I, Stark JS, et al Community fingerprinting in a sequencing world. FEMS Microbiol Ecol. 2014;89(2):316–30. 10.1111/1574-6941.12308 24580036

[22] Choe HS, Son SW, Choi HA, Kim HJ, Ahn SG, Bang JH, et al Analysis of the distribution of bacteria within urinary catheter biofilms using four different molecular techniques. Am J Infect Control. 2012;40(9):e249–54. 10.1016/j.ajic.2012.05.010 23006677

[23] Brugger SD, Frei L, Frey PM, Aebi S, Muhlemann K, Hilty M. 16S rRNA terminal restriction fragment length polymorphism for the characterization of the nasopharyngeal microbiota. PLoS One. 2012;7(12):e52241 10.1371/journal.pone.0052241 23284951 PMC3527403

[24] Castro-Carrera T, Toral PG, Frutos P, McEwan NR, Hervas G, Abecia L, et al Rumen bacterial community evaluated by 454 pyrosequencing and terminal restriction fragment length polymorphism analyses in dairy sheep fed marine algae. J Dairy Sci. 2014;97(3):1661–9. 10.3168/jds.2013-7243 24440247

[25] Jakobsson HE, Jernberg C, Andersson AF, Sjolund-Karlsson M, Jansson JK, Engstrand L. Short-term antibiotic treatment has differing long-term impacts on the human throat and gut microbiome. PLoS One. 2010;5(3):e9836 10.1371/journal.pone.0009836 20352091 PMC2844414

[26] Pilloni G, Granitsiotis MS, Engel M, Lueders T. Testing the limits of 454 pyrotag sequencing: reproducibility, quantitative assessment and comparison to T-RFLP fingerprinting of aquifer microbes. PLoS One. 2012;7(7):e40467 10.1371/journal.pone.0040467 22808168 PMC3395703

[27] Nordentoft S, Molbak L, Bjerrum L, De Vylder J, Van Immerseel F, Pedersen K. The influence of the cage system and colonisation of Salmonella Enteritidis on the microbial gut flora of laying hens studied by T-RFLP and 454 pyrosequencing. BMC Microbiol. 2011;11:187 10.1186/1471-2180-11-187 21859465 PMC3188488

[28] Lane DJ. 16S/23S rRNA sequencing In: Stackebrandt E GM, editor. Nucleic acid techniques in bacterial systematics. Chichester, United Kingdom: John Wiley and Sons; 1991 p. 115–75.

[29] Schloss PD, Westcott SL, Ryabin T, Hall JR, Hartmann M, Hollister EB, et al Introducing mothur: open-source, platform-independent, community-supported software for describing and comparing microbial communities. Appl Environ Microbiol. 2009;75(23):7537–41. 10.1128/AEM.01541-09 19801464 PMC2786419

[30] Wang Q, Garrity GM, Tiedje JM, Cole JR. Naive Bayesian classifier for rapid assignment of rRNA sequences into the new bacterial taxonomy. Appl Environ Microbiol. 2007;73(16):5261–7. 17586664 10.1128/AEM.00062-07 PMC1950982

[31] Abdo Z, Schuette UM, Bent SJ, Williams CJ, Forney LJ, Joyce P. Statistical methods for characterizing diversity of microbial communities by analysis of terminal restriction fragment length polymorphisms of 16S rRNA genes. Environ Microbiol. 2006;8(5):929–38. 16623749 10.1111/j.1462-2920.2005.00959.x

[32] Clarke KR, Warwick RM. Change in Marine Communities: An Approach to Statistical Analysis and Interpretation. 2nd edn ed. Plymouth: PRIMER-E; 2001.

[33] Bray JR, Curtis JT. An Ordination of the Upland Forest Communities of Southern Wisconsin. Ecol Monogr. 1957;27(4):325–49.

[34] Clarke KR, Somerfield PJ, Gorley RN. Testing of null hypotheses in exploratory community analyses: similarity profiles and biota-environment linkage. J Exp Mar Bio Ecol. 2008;366(1–2):56–69.

[35] Clarke KR. Non-parametric multivariate analyses of changes in community structure. Aust J Ecol. 1993;18(1):117–43.

[36] Castro-Carrera T, Frutos P, Leroux C, Chilliard Y, Hervas G, Belenguer A, et al Dietary sunflower oil modulates milk fatty acid composition without major changes in adipose and mammary tissue fatty acid profile or related gene mRNA abundance in sheep. Animal. 2015;9(4):582–91. 10.1017/S1751731114002882 25440981

[37] Hanshew AS, Jetté ME, Thibeault SL. Characterization and comparison of bacterial communities in benign vocal fold lesions. Microbiome. 2014;2:43 10.1186/2049-2618-2-43 25671105 PMC4323261

[38] Egert M, Friedrich MW. Formation of Pseudo-Terminal Restriction Fragments, a PCR-Related Bias Affecting Terminal Restriction Fragment Length Polymorphism Analysis of Microbial Community Structure. Appl Environ Microbiol. 2003;69(5):2555–62. 12732521 10.1128/AEM.69.5.2555-2562.2003 PMC154551

[39] Egert M, Friedrich MW. Post-amplification Klenow fragment treatment alleviates PCR bias caused by partially single-stranded amplicons. J Microbiol Methods. 2005;61(1):69–75. 15676197 10.1016/j.mimet.2004.11.002

[40] Rogers GB, Carroll MP, Serisier DJ, Hockey PM, Jones G, Bruce KD. characterization of bacterial community diversity in cystic fibrosis lung infections by use of 16s ribosomal DNA terminal restriction fragment length polymorphism profiling. J Clin Microbiol. 2004;42(11):5176–83. 15528712 10.1128/JCM.42.11.5176-5183.2004 PMC525137

[41] Joo S, Lee SR, Park S. Monitoring of phytoplankton community structure using terminal restriction fragment length polymorphism (T-RFLP). J Microbiol Methods. 2010;81(1):61–8. 10.1016/j.mimet.2010.01.025 20138925

[42] Metzler-Zebeli BU, Hooda S, Pieper R, Zijlstra RT, van Kessel AG, Mosenthin R, et al Nonstarch polysaccharides modulate bacterial microbiota, pathways for butyrate production, and abundance of pathogenic Escherichia coli in the pig gastrointestinal tract. Appl Environ Microbiol. 2010;76(11):3692–701. 10.1128/AEM.00257-10 20382813 PMC2876444

[43] Torok VA, Allison GE, Percy NJ, Ophel-Keller K, Hughes RJ. Influence of antimicrobial feed additives on broiler commensal posthatch gut microbiota development and performance. Appl Environ Microbiol. 2011;77(10):3380–90. 10.1128/AEM.02300-10 21441326 PMC3126468

[44] Edelman S, Leskelä S, Ron E, Apajalahti J, Korhonen TK. In vitro adhesion of an avian pathogenic Escherichia coli O78 strain to surfaces of the chicken intestinal tract and to ileal mucus. Vet Microbiol. 2003;91(1):41–56. 12441230 10.1016/s0378-1135(02)00153-0

[45] Taheri HR, Moravej H, Tabandeh F, Zaghari M, Shivazad M. Screening of lactic acid bacteria toward their selection as a source of chicken probiotic. Poult Sci. 2009;88(8):1586–93. 10.3382/ps.2009-00041 19590072

[46] Abbas Hilmi HT, Surakka A, Apajalahti J, Saris PEJ. Identification of the Most Abundant Lactobacillus Species in the Crop of 1- and 5-Week-Old Broiler Chickens. Appl Environ Microbiol. 2007;73(24):7867–73. 17933935 10.1128/AEM.01128-07 PMC2168157

[47] Bjerrum L, Engberg RM, Leser TD, Jensen BB, Finster K, Pedersen K. Microbial community composition of the ileum and cecum of broiler chickens as revealed by molecular and culture-based techniques. Poult Sci. 2006;85(7):1151–64. 16830854 10.1093/ps/85.7.1151

[48] Gong J, Si W, Forster RJ, Huang R, Yu H, Yin Y, et al 16S rRNA gene-based analysis of mucosa-associated bacterial community and phylogeny in the chicken gastrointestinal tracts: from crops to ceca. FEMS Microbiol Ecol. 2007;59(1):147–57. 17233749 10.1111/j.1574-6941.2006.00193.x

[49] Stewart EJ. Growing Unculturable Bacteria. J Bacteriol. 2012;194(16):4151–60. 10.1128/JB.00345-12 22661685 PMC3416243

[50] Vital M, Gao J, Rizzo M, Harrison T, Tiedje JM. Diet is a major factor governing the fecal butyrate-producing community structure across Mammalia, Aves and Reptilia. ISME J. 2015;9:832–43. 10.1038/ismej.2014.179 25343515 PMC4817703

[51] Komisarczuk S, Durand M, Beaumatin P, Hannequart G. Effects of phosphorus deficiency on rumen microbial activity associated with the solid and liquid phases of a fermentor (Rusitec). Reprod Nutr Dévelop. 1987;27(5):907–19. 10.1051/rnd:19870703 3685616

[52] Metzler BU, Mosenthin R, Baumgartel T, Rodehutscord M. The effect of dietary phosphorus and calcium level, phytase supplementation, and ileal infusion of pectin on the chemical composition and carbohydrase activity of fecal bacteria and the level of microbial metabolites in the gastrointestinal tract of pigs. J Anim Sci. 2008;86(7):1544–55. 10.2527/jas.2007-0267 18344312

[53] Lee N-K, Lee E-K, Paik H-D. Potential probiotic properties of phytase-producing Lactobacillus salivarius FC113. Ann Microbiol. 2013;63(2):555–60.

[54] Craven SE, Williams DD. In vitro attachment of *Salmonella typhimurium* to chicken cecal mucus: effect of cations and pretreatment with *Lactobacillus* spp. isolated from the intestinal tracts of chickens. J Food Prot. 1998;61(3):265–71. 9708293 10.4315/0362-028x-61.3.265

[55] Larsen N, Nissen P, Willats WGT. The effect of calcium ions on adhesion and competitive exclusion of *Lactobacillus* ssp. and *E*. *coli* O138. Int J Food Microbiol. 2007;114(1):113–9. 17234293 10.1016/j.ijfoodmicro.2006.10.033

[56] Metzler-Zebeli BU, Vahjen W, Baumgartel T, Rodehutscord M, Mosenthin R. Ileal microbiota of growing pigs fed different dietary calcium phosphate levels and phytase content and subjected to ileal pectin infusion. J Anim Sci. 2010;88(1):147–58. 10.2527/jas.2008-1560 19820063

[57] Geier MS, Torok VA, Allison GE, Ophel-Keller K, Hughes RJ. Indigestible carbohydrates alter the intestinal microbiota but do not influence the performance of broiler chickens. J Appl Microbiol. 2009;106(5):1540–8. 10.1111/j.1365-2672.2008.04116.x 19187131

[58] Torok VA, Ophel-Keller K, Loo M, Hughes RJ. Application of methods for identifying broiler chicken gut bacterial species linked with increased energy metabolism. Appl Environ Microbiol. 2008;74(3):783–91. 18065621 10.1128/AEM.01384-07 PMC2227708

[59] Collins RE, Rocap G. REPK: an analytical web server to select restriction endonucleases for terminal restriction fragment length polymorphism analysis. Nucleic Acids Res. 2007;35(Web Server issue):W58–62. 17631616 10.1093/nar/gkm384 PMC1933217

[60] Schütte UE, Abdo Z, Bent S, Shyu C, Williams C, Pierson J, et al Advances in the use of terminal restriction fragment length polymorphism (T-RFLP) analysis of 16S rRNA genes to characterize microbial communities. Appl Microbiol Biotechnol. 2008;80(3):365–80. 10.1007/s00253-008-1565-4 18648804

