# Correction: Spatial Variation of the Gut Microbiota in Broiler Chickens as Affected by Dietary Available Phosphorus and Assessed by T-RFLP Analysis and 454 Pyrosequencing

**DOI:** 10.1371/journal.pone.0145588

**Published:** 2015-12-17

**Authors:** Maren Witzig, Amelia Camarinha-Silva, Rebecca Green-Engert, Katharina Hoelzle, Ellen Zeller, Jana Seifert, Ludwig E. Hoelzle, Markus Rodehutscord

The second author’s name is misspelled. The correct name is: Amelia Carminha-Silva. The correction citation is: Witzig M, Camarinha-Silva A, Green-Engert R, Hoelzle K, Zeller E, Seifert J, et al. (2015) Spatial Variation of the Gut Microbiota in Broiler Chickens as Affected by Dietary Available Phosphorus and Assessed by T-RFLP Analysis and 454 Pyrosequencing. PLoS ONE 10(11): e0143442. 10.1371/journal.pone.0143442
