# Supplementary figures and images for: Spatial Variation of the Gut Microbiota in Broiler Chickens as Affected by Dietary Available Phosphorus and Assessed by T-RFLP Analysis and 454 Pyrosequencing
Source: PLoS One. 2015 Nov 20;10(11):e0143442. doi: 10.1371/journal.pone.0143442 (PMC4654470; doi:10.1371/journal.pone.0143442)

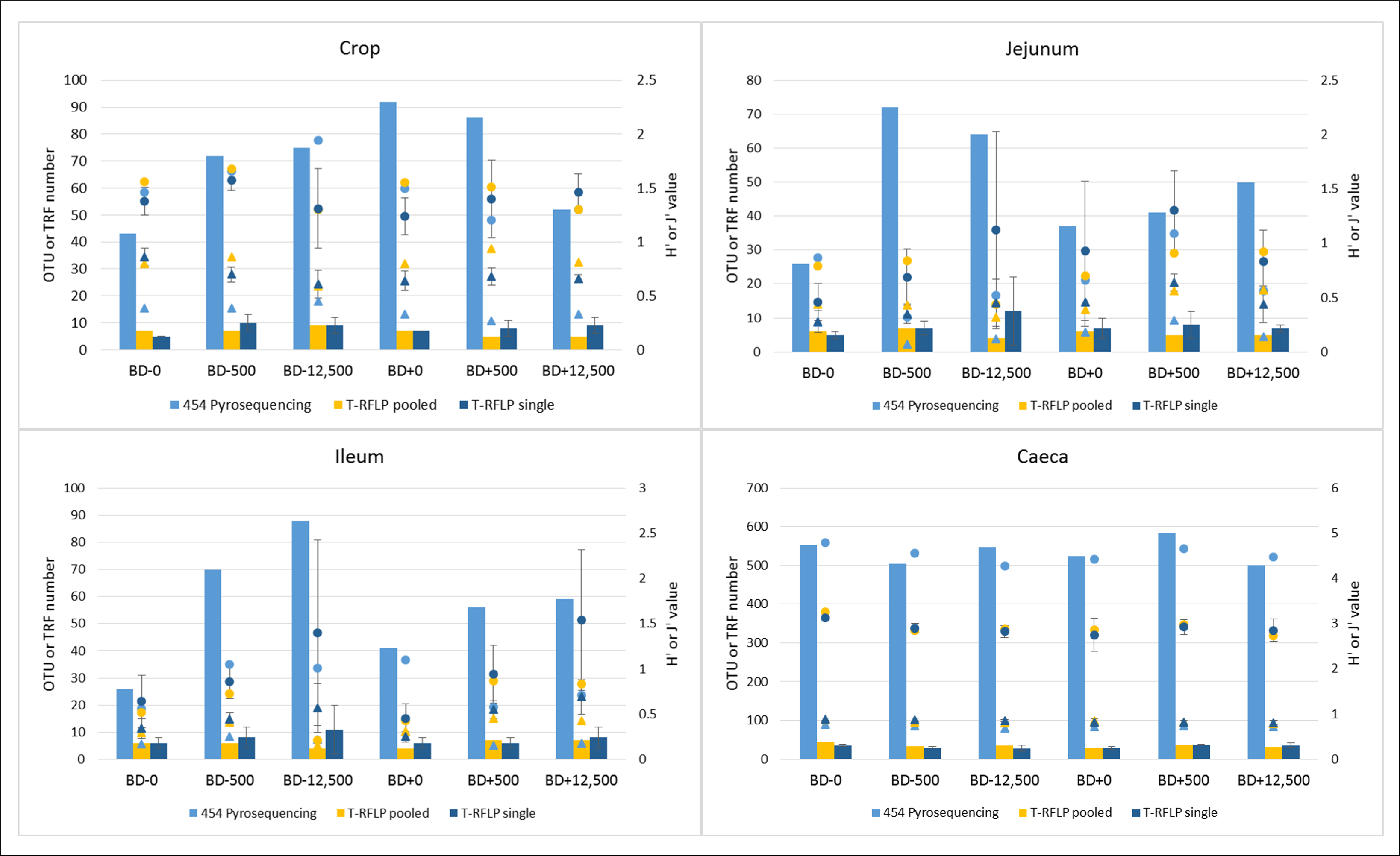

Supplement: S1 Fig — (TIFF) [file pone.0143442.s001.tiff]

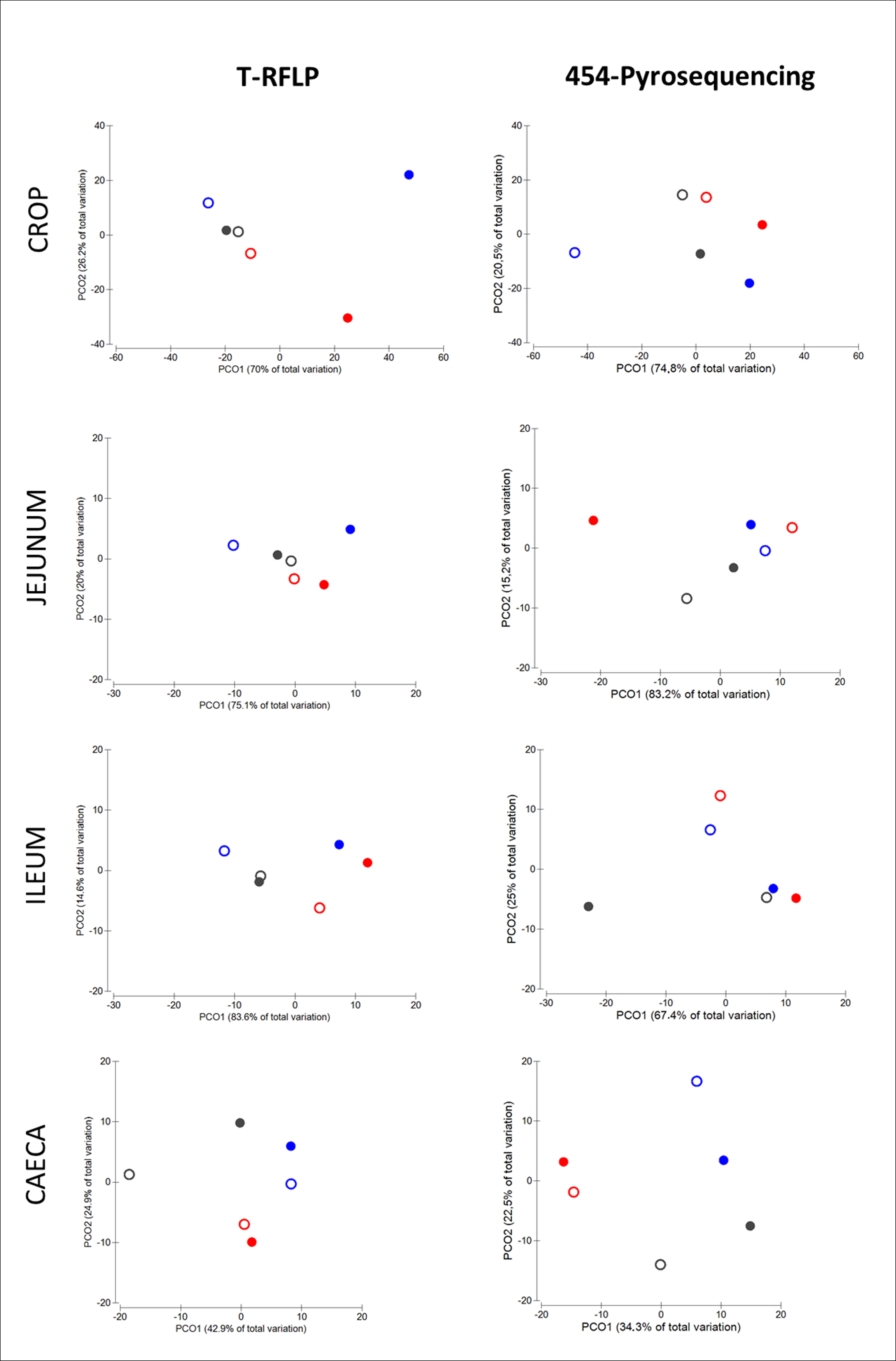

Supplement: S2 Fig — Comparison of PCoA plots for T-RFLP of pooled and 454-pyrosequencing results and samples per dietary treatment. Diets varied in supplementation of monocalcium phosphate: BD- (open circles) and BD+ (close circles) and different levels of phytase: 0 (black), 500 (red), 12,500 (blue) FTU/kg feed. (TIFF) [file pone.0143442.s002.tiff]
