# Supplementary material for: Spatial Variation of the Gut Microbiota in Broiler Chickens as Affected by Dietary Available Phosphorus and Assessed by T-RFLP Analysis and 454 Pyrosequencing
Source: PLoS One. 2015 Nov 20;10(11):e0143442. doi: 10.1371/journal.pone.0143442 (PMC4654470; doi:10.1371/journal.pone.0143442)
Supplement: S1 Table — (DOCX) [file pone.0143442.s003.docx]

**S1 Table.** **Number of operational taxonomic units (OTUs) and terminal restriction fragments (TRFs) as well as Shannon diversity (H’) and Pielous’ evenness (J’) for pooled digesta samples and single replicates (Mean, *SD*; n=3) detected for different gut sections from broiler chickens fed diets varying in monocalcium phosphate (BD-/BD+) and phytase (0, 500, 12,500 FTU/kg feed) supplementation.**

|  |  | **Pooled samples** | | | | | | **Single replicates** | | | | | |
| --- | --- | --- | --- | --- | --- | --- | --- | --- | --- | --- | --- | --- | --- |
| **Section** | **Diet** | **Pyrosequencing** | | | **T-RFLP** | | | **T-RFLP** | | | | | |
|  |  | OTUs | H’ | J’ | TRFs | H’ | J’ | TRFs | | H’ | | J’ | |
| **Crop** | BD-0 | 43 | 1.46 | 0.39 | 7 | 1.56 | 0.80 | 5 | *0* | 1.38 | *0.13* | 0.86 | *0.08* |
|  | BD-500 | 72 | 1.66 | 0.39 | 7 | 1.68 | 0.86 | 10 | *3* | 1.57 | *0.09* | 0.70 | *0.07* |
|  | BD-12,500 | 75 | 1.94 | 0.45 | 9 | 1.30 | 0.59 | 9 | *3* | 1.31 | *0.37* | 0.61 | *0.13* |
|  | BD+0 | 92 | 1.50 | 0.33 | 7 | 1.55 | 0.80 | 7 | *0* | 1.24 | *0.17* | 0.64 | *0.09* |
|  | BD+500 | 86 | 1.20 | 0.27 | 5 | 1.51 | 0.94 | 8 | *3* | 1.40 | *0.36* | 0.68 | *0.08* |
|  | BD+12,500 | 52 | 1.30 | 0.33 | 5 | 1.30 | 0.81 | 9 | *3* | 1.46 | *0.17* | 0.66 | *0.04* |
| **Jejunum** | BD-0 | 26 | 0.87 | 0.27 | 6 | 0.79 | 0.44 | 5 | *1* | 0.46 | *0.17* | 0.28 | *0.10* |
|  | BD-500 | 72 | 0.32 | 0.07 | 7 | 0.84 | 0.43 | 7 | *2* | 0.69 | *0.26* | 0.35 | *0.09* |
|  | BD-12,500 | 64 | 0.52 | 0.12 | 4 | 0.44 | 0.32 | 12 | *10* | 1.12 | *0.91* | 0.45 | *0.22* |
|  | BD+0 | 37 | 0.66 | 0.18 | 6 | 0.70 | 0.39 | 7 | *3* | 0.93 | *0.64* | 0.46 | *0.23* |
|  | BD+500 | 41 | 1.09 | 0.29 | 5 | 0.91 | 0.56 | 8 | *4* | 1.30 | *0.37* | 0.64 | *0.08* |
|  | BD+12,500 | 50 | 0.56 | 0.14 | 5 | 0.92 | 0.57 | 7 | *1* | 0.83 | *0.29* | 0.44 | *0.17* |
| **Ileum** | BD-0 | 26 | 0.56 | 0.17 | 6 | 0.52 | 0.29 | 6 | *2* | 0.64 | *0.29* | 0.34 | *0.11* |
|  | BD-500 | 70 | 1.05 | 0.25 | 6 | 0.73 | 0.41 | 8 | *4* | 0.86 | *0.19* | 0.44 | *0.07* |
|  | BD-12,500 | 88 | 1.01 | 0.22 | 4 | 0.21 | 0.15 | 11 | *9* | 1.40 | *1.03* | 0.57 | *0.27* |
|  | BD+0 | 41 | 1.10 | 0.30 | 4 | 0.43 | 0.31 | 6 | *2* | 0.45 | *0.16* | 0.25 | *0.06* |
|  | BD+500 | 56 | 0.58 | 0.15 | 7 | 0.87 | 0.45 | 6 | *2* | 0.94 | *0.32* | 0.55 | *0.10* |
|  | BD+12,500 | 59 | 0.71 | 0.18 | 7 | 0.83 | 0.43 | 8 | *4* | 1.54 | *0.78* | 0.69 | *0.19* |
| **Caeca** | BD-0 | 552 | 4.79 | 0.76 | 44 | 3.26 | 0.86 | 35 | *3* | 3.13 | *0.08* | 0.88 | *0.02* |
|  | BD-500 | 505 | 4.55 | 0.73 | 34 | 2.85 | 0.81 | 30 | *2* | 2.90 | *0.10* | 0.86 | *0.04* |
|  | BD-12,500 | 548 | 4.27 | 0.68 | 36 | 2.87 | 0.80 | 28 | *8* | 2.82 | *0.14* | 0.85 | *0.03* |
|  | BD+0 | 524 | 4.43 | 0.71 | 29 | 2.86 | 0.85 | 29 | *4* | 2.75 | *0.37* | 0.81 | *0.08* |
|  | BD+500 | 584 | 4.65 | 0.73 | 38 | 2.97 | 0.82 | 37 | *1* | 2.92 | *0.16* | 0.81 | *0.05* |
|  | BD+12,500 | 500 | 4.48 | 0.72 | 32 | 2.73 | 0.79 | 36 | *5* | 2.85 | *0.25* | 0.80 | *0.05* |
