# Supplementary material for: Spatial Variation of the Gut Microbiota in Broiler Chickens as Affected by Dietary Available Phosphorus and Assessed by T-RFLP Analysis and 454 Pyrosequencing
Source: PLoS One. 2015 Nov 20;10(11):e0143442. doi: 10.1371/journal.pone.0143442 (PMC4654470; doi:10.1371/journal.pone.0143442)
Supplement: S2 Table — (DOCX) [file pone.0143442.s004.docx]

**S2 Table. List of OTUs contributing for more than 92% of total bacteria present in the crop, jejunum and ileum and 50% in the caeca as detected by 454-pyrosequencing.**

|  |  | Relative abundance (%) | | | | | |  |  |
| --- | --- | --- | --- | --- | --- | --- | --- | --- | --- |
|  | Diet | BD-0 | BD-500 | BD-12,500 | BD+0 | BD+500 | BD+12,500 | Closest representative | RDP^a^ score |
| Crop | OTU2 | 41.0 | 40.2 | 9.9 | 55.5 | 62.4 | 69.8 | *Lactobacillus salivarius (AF089108)* | 0.92 |
|  | OTU1 | 26.3 | 13.0 | 47.3 | 20.6 | 3.0 | 5.6 | *Lactobacillus crispatus (AF257097)* | 0.92 |
|  | OTU3 | 23.7 | 30.8 | 9.9 | 10.5 | 25.8 | 8.6 | *Lactobacillus taiwanensis (EU487512)* | 0.91 |
|  | OTU10 | 3.0 | 0.8 | 12.1 | 4.7 | 1.2 | 0.6 | *Lactobacillus vaginalis (AF243177)* | 0.90 |
|  | OTU32 | 2.3 | 0.1 | 2.7 | 1.1 | 0.0 | 0.0 | *Lactobacillus oris (X94229)* | 0.87 |
|  | OTU38 | 0.2 | 2.4 | 2.1 | 0.3 | 0.3 | 0.7 | *Flavobacterium succinicans (AM230492)* | 0.93 |
|  | OTU12 | 0.2 | 6.5 | 6.0 | 1.7 | 3.4 | 4.9 | *Aeromonas sharmana (DQ013306)* | 0.82 |
|  | OTU31 | 0.0 | 1.7 | 1.9 | 1.0 | 0.6 | 1.7 | *Zoogloea oryzae (AB201043)* | 0.97 |
|  | OTU49 | 0.0 | 0.5 | 0.5 | 0.3 | 0.1 | 2.9 | *Aeromonas hydrophila (AM184287)* | 0.92 |
| Jejunum | OTU1 | 74.5 | 94.8 | 88.7 | 83.5 | 62.3 | 87.8 | *Lactobacillus crispatus (AF257097)* | 0.92 |
|  | OTU5 | 13.6 | 2.1 | 8.0 | 9.3 | 6.4 | 4.3 | *Lactobacillus aviarius (M58808)* | 0.87 |
|  | OTU3 | 7.7 | 1.2 | 1.1 | 3.5 | 21.9 | 1.4 | *Lactobacillus taiwanensis (EU487512)* | 0.91 |
|  | OTU2 | 3.0 | 0.7 | 0.4 | 2.7 | 8.4 | 5.3 | *Lactobacillus salivarius (AF089108)* | 0.92 |
| Ileum | OTU1 | 87.9 | 68.9 | 75.2 | 62.0 | 87.8 | 84.3 | *Lactobacillus crispatus (AF257097)* | 0.92 |
|  | OTU5 | 5.4 | 18.2 | 14.0 | 6.0 | 1.1 | 3.0 | *Lactobacillus aviarius (M58808)* | 0.87 |
|  | OTU2 | 3.5 | 7.3 | 2.4 | 1.4 | 6.8 | 8.2 | *Lactobacillus salivarius (AF089108)* | 0.92 |
|  | OTU3 | 1.7 | 2.4 | 0.4 | 0.3 | 0.7 | 0.7 | *Lactobacillus taiwanensis (EU487512)* | 0.91 |
|  | OTU6 | 0.4 | 1.0 | 3.9 | 26.9 | 0.1 | 1.3 | *Lactobacillus aviarius (M58808)* | 0.89 |
| Caeca | OTU4 | 10.8 | 17.0 | 21.6 | 15.1 | 17.8 | 18.9 | *Bacteroides fragilis (CR626927)* | 0.96 |
|  | OTU6 | 4.5 | 0.7 | 4.1 | 0.8 | 0.4 | 0.3 | *Lactobacillus aviarius (M58808)* | 0.89 |
|  | OTU7 | 3.2 | 2.8 | 2.7 | 10.2 | 1.7 | 4.2 | *Unc. Firmicutes bacterium (FJ440067)* | 0.87 |
|  | OTU8 | 4.0 | 1.9 | 5.8 | 3.4 | 1.5 | 4.0 | *Alistipes finegoldii (AY643083)* | 0.90 |
|  | OTU9 | 2.6 | 3.6 | 3.0 | 2.8 | 2.7 | 2.8 | *Unc. chicken cecal bacterium (AB075613)* | 0.86 |
|  | OTU11 | 3.1 | 3.7 | 1.7 | 2.6 | 4.0 | 1.6 | *Bacteroides ovatus (AB050108)* | 0.80 |
|  | OTU13 | 2.5 | 4.2 | 2.0 | 1.3 | 4.5 | 1.2 | *Unc. bacterium (FJ367899)* | 0.89 |
|  | OTU14 | 1.9 | 3.4 | 1.6 | 2.0 | 1.5 | 2.2 | *Clostridium orbiscindens (Y18187)* | 0.82 |
|  | OTU15 | 1.2 | 1.2 | 2.2 | 1.0 | 3.8 | 1.5 | *Unc. Firmicutes bacterium (FJ440067)* | 0.74 |
|  | OTU16 | 1.0 | 0.0 | 0.0 | 4.9 | 0.0 | 3.8 | *Bacteroides thetaiotaomicron (AE015928)* | 0.91 |
|  | OTU17 | 2.3 | 0.9 | 1.1 | 1.2 | 2.3 | 1.6 | *Unc. bacterium (EU009838)* | 0.91 |
|  | OTU18 | 2.1 | 0.7 | 2.3 | 1.7 | 0.7 | 1.0 | *Unc. bacterium (EF025290)* | 0.88 |
|  | OTU19 | 0.7 | 0.6 | 1.6 | 1.5 | 0.6 | 2.1 | *Unc. bacterium (DQ532165)* | 0.88 |
|  | OTU20 | 1.1 | 1.7 | 1.4 | 0.7 | 1.2 | 0.7 | *Unc. bacterium (EF025253)* | 0.97 |
|  | OTU21 | 1.0 | 0.7 | 1.4 | 1.4 | 0.9 | 1.2 | *Clostridium spiroforme (X75908)* | 0.86 |
|  | OTU22 | 1.5 | 1.7 | 0.6 | 0.6 | 0.9 | 1.6 | *Unc. bacterium (GQ175453)* | 0.92 |
|  | OTU23 | 0.9 | 2.0 | 0.7 | 1.1 | 0.8 | 0.7 | *Unc. bacterium (FJ507937)* | 0.95 |
|  | OTU25 | 0.9 | 2.9 | 0.5 | 0.4 | 0.8 | 0.6 | *Unc. bacterium (HQ716358)* | 0.75 |
|  | OTU26 | 1.5 | 1.3 | 1.1 | 0.5 | 0.8 | 0.2 | *Unc. bacterium (AM694064)* | 0.76 |
|  | OTU27 | 0.7 | 0.3 | 1.2 | 0.3 | 0.7 | 1.9 | *Unc. bacterium (GU171133)* | 0.94 |
|  | OTU28 | 1.0 | 0.2 | 1.3 | 1.2 | 0.6 | 0.6 | *Unc. bacterium (DQ455830)* | 0.99 |
|  | OTU29 | 1.6 | 0.1 | 0.5 | 1.7 | 0.0 | 1.3 | *Unc. bacterium (JQ208287)* | 0.72 |
|  | OTU30 | 1.3 | 1.6 | 0.3 | 1.1 | 0.7 | 0.4 | *Unc. organism (HQ807030)* | 0.88 |
|  | OTU33 | 1.0 | 0.3 | 0.3 | 1.5 | 0.2 | 1.4 | *Helicobacter canadensis (AF262037)* | 0.92 |
|  | OTU35 | 0.4 | 0.9 | 0.8 | 0.8 | 1.2 | 0.4 | *Eubacterium desmolans (L34618)* | 0.75 |
|  | OTU41 | 0.2 | 0.4 | 1.9 | 0.6 | 0.1 | 0.4 | *Unc. bacterium (DQ804044)* | 0.75 |

^a^Ribosomal database project
