# Supplementary material for: Spatial Variation of the Gut Microbiota in Broiler Chickens as Affected by Dietary Available Phosphorus and Assessed by T-RFLP Analysis and 454 Pyrosequencing
Source: PLoS One. 2015 Nov 20;10(11):e0143442. doi: 10.1371/journal.pone.0143442 (PMC4654470; doi:10.1371/journal.pone.0143442)
Supplement: S3 Table — (DOCX) [file pone.0143442.s005.docx]

**S3 Table. Positive and negative correlations within crop, jejunum, ileum and caeca.**

|  |  |  | Pearson | p-value |
| --- | --- | --- | --- | --- |
| Crop | *L. crispatus* | *L. vaginalis* | 0.93 | 0.007 |
|  | *L. crispatus* | *L. reuteri* | 0.97 | 0.001 |
|  | *L. vaginalis* | *L. reuteri* | 0.85 | 0.01 |
|  | *L. salivarius* | *L. crispatus* | -0.91 | 0.01 |
|  | *L. salivarius* | *L. vaginalis* | -0.82 | 0.04 |
|  | *L. salivarius* | *L. reuteri* | -0.92 | 0.01 |
| Jejunum | *L. reuteri* | *L. taiwanensis* | 0.97 | 0.001 |
|  | *L. crispatus* | *L. reuteri* | -0.92 | 0.08 |
|  | *L. crispatus* | *L. taiwanensis* | -0.95 | 0.004 |
| Ileum | *L. reuteri* | *L. taiwanensis* | 0.90 | 0.01 |
|  | *L. salivarius* | *L. vaginalis* | -0.97 | 0.001 |
