# Supplementary material for: Spatial Variation of the Gut Microbiota in Broiler Chickens as Affected by Dietary Available Phosphorus and Assessed by T-RFLP Analysis and 454 Pyrosequencing
Source: PLoS One. 2015 Nov 20;10(11):e0143442. doi: 10.1371/journal.pone.0143442 (PMC4654470; doi:10.1371/journal.pone.0143442)
Supplement: S4 Table — Diets differed in supplementation of monocalcium phosphate (BD-/BD+) and phytase (0, 500, 12,500 FTU/kg feed). (DOCX) [file pone.0143442.s006.docx]

**S4 Table. Relative abundance (%) of bacterial families within the crop, jejunum, ileum and caeca.**

| **Section** | **Family** | **BD-**  **0** | **BD-**  **500** | **BD- 12,500** | **BD+**  **0** | **BD+ 500** | **BD+ 12,500** |
| --- | --- | --- | --- | --- | --- | --- | --- |
| **Crop** | *Aeromonadaceae* | 0.19 | 7.13 | 6.55 | 2.03 | 3.55 | 7.80 |
|  | *Flavobacteriaceae* | 0.23 | 2.78 | 2.97 | 0.57 | 0.40 | 1.27 |
|  | *Lactobacillaceae* | 98.99 | 86.42 | 85.04 | 94.67 | 93.11 | 85.18 |
|  | *Rhodocyclaceae* | 0.04 | 1.71 | 1.89 | 1.07 | 0.78 | 1.79 |
|  | Others | 0.56 | 1.96 | 3.55 | 1.65 | 2.16 | 3.96 |
| **Jejunum** | *Lactobacillaceae* | 99.94 | 99.64 | 99.63 | 99.83 | 99.78 | 99.89 |
|  | Others | 0.06 | 0.36 | 0.37 | 0.17 | 0.22 | 0.11 |
| **Ileum** | *Enterobacteriaceae* | 0.43 | 1.05 | 3.91 | 27.85 | 0.12 | 1.40 |
|  | *Enterococcaceae* | 0 | 0.02 | 0.79 | 1.05 | 0.06 | 0.38 |
|  | *Lactobacillaceae* | 99.54 | 97.66 | 92.91 | 70.39 | 99.59 | 97.45 |
|  | *Peptostreptococcaceae* | 0.01 | 0.37 | 0.46 | 0.09 | 0.05 | 0.41 |
|  | Others | 0.02 | 0.89 | 1.93 | 0.62 | 0.19 | 0.37 |
| **Caeca** | *Bacteroidaceae* | 14.87 | 20.69 | 23.40 | 22.70 | 21.86 | 24.42 |
|  | *Erysipelotrichaceae* | 3.90 | 2.74 | 2.76 | 4.29 | 2.42 | 2.99 |
|  | *Enterobacteriaceae* | 4.54 | 0.73 | 4.14 | 0.78 | 0.37 | 0.27 |
|  | *Helicobacteraceae* | 1.00 | 0.33 | 0.31 | 1.55 | 0.23 | 1.44 |
|  | *Lachnospiraceae* | 10.13 | 8.57 | 7.56 | 7.48 | 8.72 | 6.42 |
|  | *Rikenellaceae* | 3.95 | 1.93 | 5.78 | 3.41 | 1.53 | 3.95 |
|  | *Ruminococcaceae* | 41.00 | 43.53 | 37.34 | 36.35 | 43.13 | 40.39 |
|  | *Unc. Clostridiales* | 10.73 | 10.20 | 11.30 | 16.56 | 13.28 | 12.50 |
|  | Others | 9.88 | 11.28 | 7.40 | 6.89 | 8.47 | 7.62 |
